# Overcome Drug Resistance in Cholangiocarcinoma: New Insight Into Mechanisms and Refining the Preclinical Experiment Models

**DOI:** 10.3389/fonc.2022.850732

**Published:** 2022-03-17

**Authors:** Qingfan Zheng, Bin Zhang, Changfeng Li, Xuewen Zhang

**Affiliations:** ^1^ Department of Hepatobiliary and Pancreas Surgery, the Second Hospital of Jilin University, Changchun, China; ^2^ Department of Endoscopy Center, China-Japan Union Hospital of Jilin University, Changchun, China

**Keywords:** cholangiocarcinoma, research model, drug resistance, mechanisms, patient-derived organoids

## Abstract

Cholangiocarcinoma (CCA) is an aggressive tumor characterized by a poor prognosis. Therapeutic options are limited in patients with advanced stage of CCA, as a result of the intrinsic or acquired resistance to currently available chemotherapeutic agents, and the lack of new drugs entering into clinical application. The challenge in translating basic research to the clinical setting, caused by preclinical models not being able to recapitulate the tumor characteristics of the patient, seems to be an important reason for the lack of effective and specific therapies for CCA. So, there seems to be two ways to improve patient outcomes. The first one is developing the combination therapies based on a better understanding of the mechanisms contributing to the resistance to currently available chemotherapeutic agents. The second one is developing novel preclinical experimental models that better recapitulate the genetic and histopathological features of the primary tumor, facilitating the screening of new drugs for CCA patients. In this review, we discussed the evidence implicating the mechanisms underlying treatment resistance to currently investigated drugs, and the development of preclinical experiment models for CCA.

## Introduction

Cholangiocarcinoma (CCA) is a kind of “biliary tract cancer” and can be subdivided into intrahepatic CCA (iCCA), perihilar CCA (pCCA), and distal CCA (dCCA) according to the anatomic location. CCA development may be associated with several risk factors, such as opisthorchis viverrini or clonorchis sinensis infection (1), hepatolithiasis (2), primary sclerosing cholangitis (PSC) (3), viral hepatitis and cirrhosis (4) and genetic reasons (5). CCA ranks second in the frequency of primary liver cancer and is characterized by rapid disease progression and dismal prognosis (6). The 5-year survival rate is 30% in patients with early-stage, 24% in patients with regional lymph nodes metastasis and 2% in patients with distant metastasis (7). Increasing studies have suggested that the incidence of CCA is increasing all around the world (8), which makes CCA a growing health concern. Surgical resection is the only curative treatment in CCA; however, more than 70% of patients are diagnosed with the unresectable disease as a result of clinical silence (9), and patients receiving surgery also have a high frequency of relapse and metastasis. Systemic therapies, such as cytotoxic chemotherapy, radiotherapy, targeted therapy, and immunotherapy, have been applied to improve the prognosis of CCA patients. Based on the result of the ABC-02 trial, patients with unresectable CCAs are recommended to receive a combination treatment of cisplatin and gemcitabine (10). However, the long-term prognosis of CCA patients treated with cisplatin and gemcitabine remains to be poor as a result of drug resistance (11).

Unfortunately, few more compounds have been approved for CCA treatment. The shortage of therapeutic strategies in CCA might be attributed to two main reasons. The first one is that the poor preclinical models fail to represent the multiple features of the tumor of the patient, leading to the dilemma that many drugs which show well anti-tumor efficiency in preclinical research finally fail in clinical trials (12, 13). CCA is characterized by pronounced multilevel intertumoral heterogeneity and the complete molecular landscape of CCA remains to be elusive, limiting the development of effective target therapies in CCA. Novel preclinical research models, recapitulating CCA heterogeneity, may contribute to screening effective drugs for CCA patients. Recently, patient-derived tumor xenografts (PDXs) and patient-derived tumor organoids (PDOs) have become the hotspot. They are regarded as preferable cancer models to investigate the sensitivity of anticancer drugs, for their advantage of better mimicking the biology of primary tumors than cell line models.

The second reason for the drug shortage in CCA treatment is the developed drug resistance. For these drugs going beyond clinical studies, limited benefits were observed mainly because of intrinsic or acquired treatment resistance in CCA. A better understanding of the mechanisms underlying drug resistance in CCA is meaningful for developing novel therapeutic strategies for the treatment of CCA. Great efforts have been devoted to investigating the mechanisms contributing to the drug resistance of CCAs. CCAs could downregulate pro-apoptotic regulators (caspase-3 and caspase-9) to overcome chemotherapy-induced death (14). Diosgenin derivates might augment the chemotherapy sensitivity in CCA through targeting caspase-3 and -9 (15). When treated with FGFR inhibitors, CCAs also undergo secondary FGFR mutation to acquire resistance (16). The tumor microenvironment (TME) also contributes to drug resistance in CCA (17). However, that is probably just the tip of the iceberg. Better cancer models and better understandings of the underlying mechanisms contributing to treatment resistance will facilitate the development of strategies counteracting drug resistance in CCA.

Herein, we review the current development of experimental models in the studies of drug sensitivity in CCA and evidence implicating the mechanisms underlying treatment resistance to currently investigated drugs, hoping to provide new insights into developing new therapeutic targets or treatment strategies to improve the prognosis of patients with CCA.

## Experiment Models for Drug Screening in CCA

As a result of the increasing incidence of CCA worldwide and the dismal prognosis of patients, new therapeutic strategies for CCA are urgently needed. In this regard, ideal preclinical models, which can facilitate our understanding of heterogenicity within CCA and molecular mechanisms responsible for CCA development, will pave the way for identifying new therapeutic targets and determining the effectiveness of therapies through high throughput experiments. Next, we will review the development of experimental models for CCA.

### Two-Dimension Culture Models for CCA

CCA cell lines are the most commonly used and well-characterized experimental model. During the last three decades, more than fifty CCA cell lines have been established after the first one was established in 1985 (18, 19). The mostly frequently used cell lines and their characteristics are summarized (**Table 1**). Cell lines are characterized by being easy to maintain, undergoing genetic modification and providing reproducible and fast results (36). A generally used method to investigate the mechanisms for treatment resistance is analyzing differentially expressed genes between the wild-type cell lines and drug-resistant cell lines, generated through undergoing repeat and increasing treatment of drug. Genetic manipulation, such as gene overexpression, knockdown or point mutation can also be used to establish drug-resistant cell lines. Cell line model is very easy to conduct gene editing, so CRISPR libraries or RNA interference (RNAi) libraries are highly useful in screening genes implicated in drug resistance in cell line model (37). Using cell line models, more than 100 genes have been identified to be associated with drug resistance in CCA (38, 39), such as multidrug resistance protein 3 (MRP3/ABCC3) in the plasma membrane (40), Bcl2 in mitochondria (41). Cell line models have contributed greatly to investigating molecular mechanisms critical in CCA progression and developing strategies to overcome drug resistance. However, it has been revealed recently that the clinical translational value of cell lines is poor. Despite that great efforts have been devoted to developing new targeted therapies based on positive results generated from cell line experiment models, it is pitiful that few compounds go beyond preclinical study, which may be mainly attributed to failing to recapitulate the multiple features of the tumor of the patient, leading to the dilemma that many drugs which show well anti-tumor efficiency in cell line models finally fail in clinical trials (12, 13). To overcome some drawbacks of cell line models, a two-dimensional primary culture model for CCA was established (42, 43). Primary CCA cultures can be established using surgically resected tissue, and it can better recapitulate the genetic properties of cancer tissue from CCA patients (44). Using the primary culture model, Fraveto et al. discovered the differences between mucin- and mixed-type CCA in terms of sensitivity to chemotherapeutic and molecular targeted agents (45). However, there are several apparent drawbacks of two-dimensional primary culture, such as only being feasible in patients receiving surgery, the genetic adaption to selective pressure in a two-dimension culture model and not being able to evaluate the effect of tumor microenvironment on drug sensitivity (46).

**Table 1:** The most frequently used CCA cell lines and their characteristics.

Cell line	Anatomic site	Source	Drug sensitivity (IC50: half maximal inhibitory concentration)	Genetic alteration	Reference
HuH28	iCCA	Primary tumor	Erlotinib: resistant, IC50 >10 μM; Gefitinib: resistant, IC50 >10 μM; Sorafenib: resistant, IC50 >10 μM; Lapatinib: sensitive, IC50 = 2.02 μM; Trametinib: resistant, IC50 >50 nM; Panitumumab, resistant, IC50 >5 μM.	mPIK3CA; mTP53	(20–23)
HuCCT1	iCCA	Metastasis (Ascites)	Gemcitabine: sensitive, IC50 = 670 nM; Cisplatin: resistant, IC50 >10 μM; Olaparib: sensitive, IC50 = 68nM; FGFR inhibitor BGJ-398:resistant; apatinib: IC50 = 8 μM; Autophagy/PPTI inhibitor GSN561:sensitive, IC50 = 1.5 μM.	mKRAS; mTP53; BAP1↑	(24–29)
KMC-1	iCCA	Primary tumor	NA	mBRAF; mPTEN; mEGF	(30)
RBE	iCCA	Primary tumor	FGFR inhibitor BGJ-398:resistant; Anlotinib: IC50 = 4.67 μM (72 h); gemcitabine:sensitive, IC50 = 0.03 μM (72 h); apatinib: IC50 = 8 μM Autophagy/PPTI inhibitor GSN561:sensitive, IC50 = 1.7 μM; 5-Fu: resistant, IC50 >1 mM.	mIDH1; mBIRC6; mKRAS	(24, 28, 29, 31, 32)
EGI-1	eCCA/dCCA	Primary tumor	Erlotinib: IC50 = 5.72 μM; Gefitinib: IC50 = 2.48 μM; Sorafenib: IC50 = 2.06 μM; Lapatinib: IC50 = 4.20 μM; Trametinib: sensitive, IC50 = 6.25 nM; Panitumumab, resistant, IC50 >5 μM.	mKRAS; mTP53	(22, 23)
TFK1	eCCA/dCCA	Primary tumor	Erlotinib: IC50 = 2.59 μM; Gefitinib: IC50 = 1.8 μM; Sorafenib: IC50 = 6.2 μM; Lapatinib: IC50 = 5.25 μM; Trametinib: resistant, IC50 >50 nM; Panitumumab, resistant, IC50 >5 μM.	mTP53; mMSH6	(22, 23, 33)
HCCC-9810	iCCA	Primary tumor	Anlotinib: IC50 = 8.13 μM (72 h); gemcitabine: sensitive, IC50 = 0.28 μM (72 h); 5-Fu: resistant, IC50 >2.5 mM.	Not available	(31, 32)
CCLP1	iCCA	Primary tumor	FGFR inhibitor BGJ-398:sensitive, IC50 2–15 nM.	mTP53; mBAP1; mCTNNB1	(26)
QBC939	eCCA	Primary tumor	gemcitabine: sensitive, IC50 = 1.2 μM; Cisplatin: resistant, IC50 >20 μM; 5-Fu: resistant, IC50 >40 μM.	Not available	(29, 34, 35)

### Three-Dimension Culture Models for CCA

**Figure 1:**
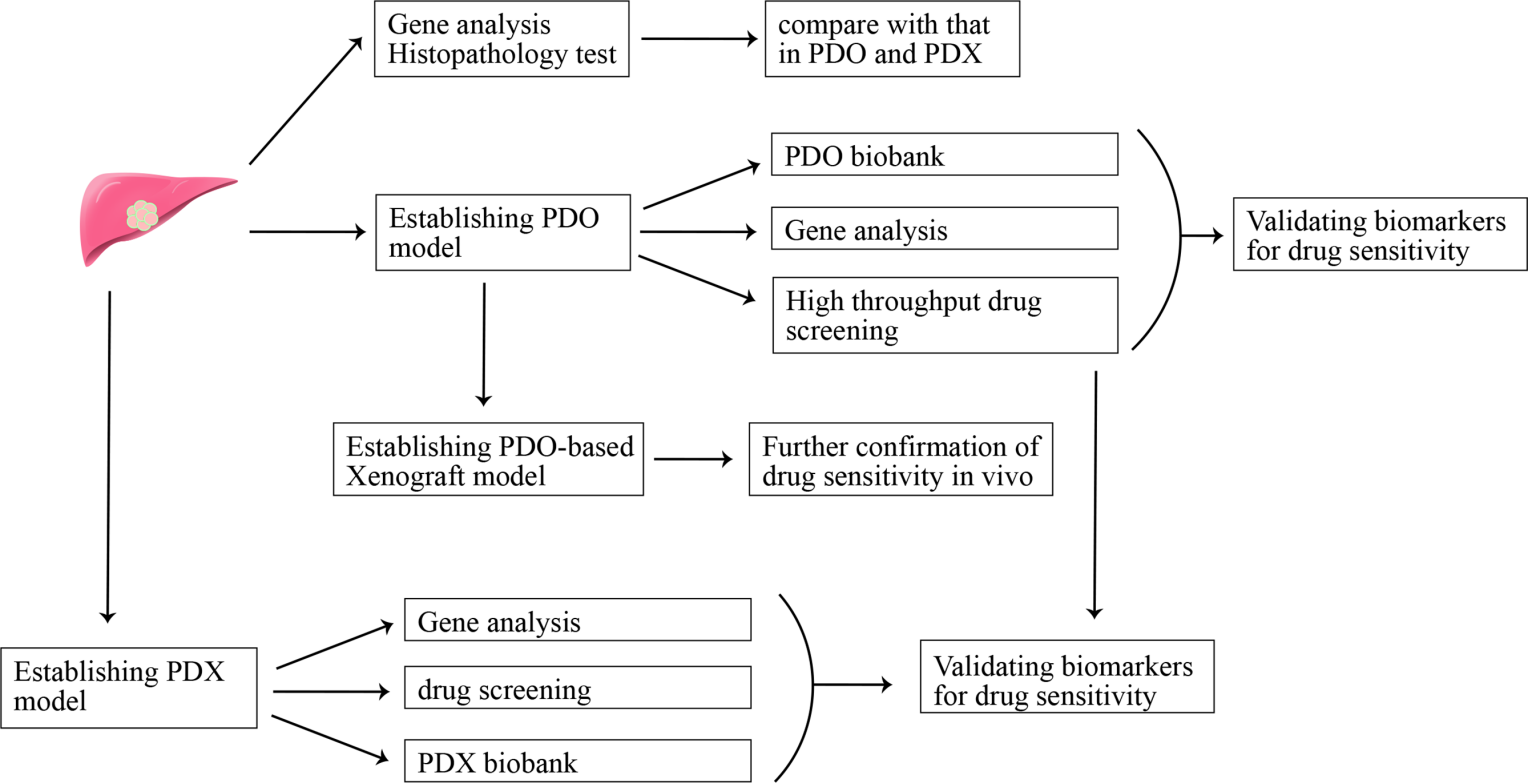
Patient-derived organoid (PDO) model and patient-derived xenograft (PDX) model in precision medicine.

To further mimic real tumor tissues where cancer cells tightly interact with each other, three-dimension culture systems were established (47). The spheroid-forming assay is a common three-dimension culture model for investigating stemness properties of cancer cells. Cancer has been regarded as a heterogeneous population of cells, and there is a small percentage of tumor cells being called cancer stem-like cells (CSCs) for their ability of self-renewing and multi-lineage differentiation. Recently, CSCs are regarded to be significant contributors to chemotherapy resistance and tumor relapse (48, 49). The sphere-forming assay is a common method to enrich CSCs. CCA spheroids can be established through suspension culture of cell lines or freshly isolated single cells from tissues of CCA patients in a serum-free culture medium supplemented with growth factors (50–52). Different from a two-dimension culture where cancer cells quickly obtain a phenotype of differentiation, spheroid cells exhibit increased expression of stemness-associated genes (e.g., OCT4, Nanog, BMI1, CD133, Lgr5) and possess stemness properties, such as persistent self-renewing, enhanced tumorigenicity and resistance to chemotherapeutic compounds (53). This model can be used to decipher the mechanism for CSCs-mediated drug resistance. However, spheroids cannot represent the heterogeneous population of cells in tumors. Recently, the development of single-cell RNA-seq technology facilitated our understanding of the heterogeneous property of tumors, and heterogenous is thought to be closely associated with drug resistance (54). So, establishing preclinical experimental models recapitulating the heterogenous property of tumor may pave the way for screening drugs for CCA patients. There are high hopes for another kind of three-dimension *in vitro* culture model-organoid and patient-derived xenograft (PDX) *in vivo* models to break this tension (**Figure 1**). The latter one will be discussed in the next part “*in vivo* models for CCA”.

Tumor organoid refers to self-organizing organotypic structures formed by tissue-derived adult stem cells when cultured in a basement membrane‐mimicking hydrogel and organoid-specific culture medium (55). Organoid has become a hotspot for their advantage of recapitulating the heterogeneity of original tumors, showing promising potential in facilitating translation from basic to clinical research (55). A recent study evaluated the potential of organoids in predicting clinical outcomes in patients (56). The conclusion that organoids can be used as a model to predict patient response to anti-cancer agents in the clinic was drawn after comparing sensitivity results of drug screening in organoid and organoid-based mouse tumor xenograft model with that of patients in clinical trials. In addition, organoids may help identify new biomarkers for predicting treatment response through matching molecular profiling of tumor organoids to drug screening results (56). Recently, CCA organoid has been reported to be established from freshly resected specimens and core needle biopsies (57, 58). The CCA organoid culture medium reported in these studies is comprised of advanced DMEM/F-12, B-27, N-2, nicotinamide, N-acetyl-L-cysteine, [Leu15]-gastrin, forskolin, A83-01, EGF, FGF10, HGF, RSpo1 and Wnt3a (58). CCA organoids highly resemble the patient tumor in terms of histopathology and genetic mutation, even upon being transplanted into immune-deficient mice after a short time of *in vitro* three-dimension culture. An organoid-based drug screening system is established to detect their sensitivity to 29 anti-cancer compounds, and the result can provide a reference for personalized medicine application in CCA patients (58). CCA organoids can also be established *via* engineering genetic mutations-mediated transformation of normal organoids (59). This model provides a platform to evaluate the role of specific genes in cancer initiation, progression, and drug resistance. Clinical trials evaluating the sensitivity of using organoids to predict drug response in CCA patients will further highlight the value of organoids in clinical application, and organoid model-based drug screening may promote the development of precision medicine in CCA.

### *In Vivo* Models for CCA

An apparent disadvantage of *in vitro* culture model for CCA research is lacking the complex tumor microenvironment (TME), and this can be rectified by *in vivo* mouse models. For example, paclitaxel and nanoparticle albumin-bound paclitaxel exhibited similar anti-tumor effect in *in vitro* experiment; whereas only nanoparticle albumin-bound paclitaxel could inhibit tumor growth in *in vivo* models, for its ability to reverse CAF-mediated paclitaxel resistance, offering evidence for the advantage of *in vivo* model in studying the role of TME in drug resistance (60). The most used *in vivo* model for screening novel anti-cancer agents in CCA is the xenotransplant mouse model of human CCA cell lines, where human CCA cell lines are injected into the athymic nude or severe combined immune-deficient (SCID) mice (61). Neoplastic cells can be injected subcutaneously (subcutaneous implantation) or into the liver (orthotopic implantation). The xenograft model may only represent CCA at the advanced stage. Two other *in vivo* models-transgenic genetically‐engineered mouse models and carcinogens-induced CCA mouse models may recapitulate the progress of tumor initiation and progression and provide an opportunity to analyze drug sensitivity in different cancer stages. These two models are generated in immunocompetent mice, so they can be used to study the effect of immune microenvironment on the malignant phenotype of CCA, and the immunotherapy response in CCA. The most frequently used animal models for CCA and their characteristics were summarized (**Table 2**). The transgenic genetically‐engineered mouse models and carcinogen-induced CCA mouse models have been extensively reviewed elsewhere (71), we only briefly introduce their characteristics in **Table 2**, and we will not discuss it in detail here.

**Table 2:** Animal models frequently used in CCA and their characteristics.

Model name	Generation	Characteristics	Advantages	Disadvantages
Cell line-based Xenograft models	Cell lines got transplanted into mice (Subcutaneously or Orthotopically)	Imitate tumors in advanced stage.	Short experimental cycle; Low cost; Most commonly used;	No immune microenvironment
Patient-derived Xenograft models	Patients’ tissue got transplanted into mice (Subcutaneously or Orthotopically)	Imitate tumors in advanced stage.	Recapitulate the heterogeneity of tumor	No immune microenvironment;
Genetically engineered mice (GEM) model/Reference				
Smad4-Pten model (62)	Smad4co and PTENco with Alb-cre mice	Imitate tumor at different stage. Bile duct hyperplasia at 2 months; Tumor development at 4–7 months	Similar to human iCCA;	Mixed HCC-CCA phenotype; No inflammation; No chronic liver injury; No metastases
Kras-IDH model (63)	mIDH2, mKRAS with Alb-Cre mice.	Imitate tumor at different stage; tumors appear at 33–58 weeks	Similar to human iCCA; Spontaneous metastases	Long latency time
KRas-Pten model (64)	mKRas, PTENflox with alb-Cre mice	Imitate tumor at different stage; Multiple tumor nodules	Similar to human iCCA; Short tumor latency	No chronic liver injury; No metastases. No inflammation
KRas-P53 model (65)	mKrasG12D, p53 deletion with alb-Cre mice	Imitate tumor at different stage; Tumors appears at 9 weeks of age	Similar to human iCCA; Spontaneous metastases	No chronic liver injury; No inflammation;
ErbB model (66)	Bovine Keratin 5 (BK5) promoter-mediated constitutive expression of ErbB2.	Imitate tumor at different stage; iCCA appears at 4 months	Similar to human iCCA;	Long latency time Gallbladder carcinoma model;
Notch1 model (67)	Alb-Cre mice with constitutive overexpression of Notch1	Imitate tumor at different stage;	Homoplastic transplantation	Mixed HCC-CCA phenotyp; Long latency time
Tp53^−/−^ CCl4 model (62)	Tp53^−/−^ mice treated with CCl4	Imitate tumor at different stage; iCCA development in 54% of mice. Injury and fibrosis in bile duct after 4 months of treatment	Chronic liver injury; fibrosis and inflammation	Development of HCC; Long treatment with CCl4
Hydrodynamic Tail Vein Injection (HTVI) Models/Reference				
Yap and PI3KCA model (68)	Sleeping Beauty transposon toolbox, Yap and PI3KCA plasmid Injected into wt mice	Imitate tumor at different stage;	iCCAs cover ~80% of the liver parenchyma within 12–13 weeks post-HTVI	Mixed HCC-CCA phenotype
NICD1 and AKT model (69)	Sleeping Beauty transposon toolbox, NCID1 and Akt plasmid Injected into wt mice	Imitate tumor at different stage; iCCA appears 4.5 weeks after HTVI	Similar to human iCCA	Not mentioned
AKT and YAP model (70)	Sleeping Beauty transposon toolbox, AKT and YAP plasmid Injected into wt mice	Imitate tumor at different stage;	Similar to human iCCA	Relatively low successful rate

The subcutaneous xenograft model of CCA cell lines has been widely applied in studying molecular mechanisms contributing to CCA progression and drug resistance (61, 72). Generally, CCA cell lines are injected into the back flank of SCID mice, and the mice are treated with anti-cancer agents after the formation of tumor mass, then the effects of anti-cancer agents on tumor growth are observed (73). Real-time monitoring of tumor growth can be achieved through directly measuring tumor volume by a caliper (74). In a representative study of this model, mice are subcutaneously injected with Sk-ChA-1 cells and treated with tamoxifen after the formation of CCA xenografts. The result reveals that Tamoxifen can significantly inhibit CCA growth *in vivo* (75). When injected with genetically manipulated cells, CCA subcutaneous xenograft models can also be used to evaluate gene-determined drug sensitivity by comparing tumor growth between wild-type cells and genetically manipulated cells under the effect of anti-cancer drugs. Compared with the subcutaneous xenograft model of CCA, orthotopic xenograft models may generate a TME more like original CCA tissue, favoring spontaneous metastasis of CCA cells. In addition, orthotopic models better recapitulate pharmacodynamic properties in humans and predict clinical therapeutic outcomes more accurately (74). The development of *in vivo* cancer imagining technology allows the real-time imagine of tumor growth in orthotopic models (76). The establishment of orthotopic models for CCA is more technocratic. CCA cells are seeded into the liver parenchyma directly (77) or delivered through the portal (78) or splenic vein (79). Orthotopic xenograft can also be established through implanting small fragments of tumor mass subcutaneously grown in donor mice (80). Gene therapies overcoming sorafenib resistance in CCA were evaluated using this model (80). Metastasis is an important determiner for the poor prognosis of patients, therapies targeting tumor metastasis are needed to improve the outcome of patients. The orthotopic xenograft models can also be used to evaluate the effect of anti-cancer agents on the metastasis of the CCA cell line *in vivo* (81).

Recently, CCA PDX models have been established by engrafting patient tumor tissue or patient tumor tissue-generated organoid into immunocompromised mice. It is regarded to be the most clinic-resembling *in vivo* model for retaining key characteristics of original tumor cells and recapitulating the drug response of human cancer to anti-cancer agents. Cavalloni et al. established the first PDX model of CCA with KRAS mutation (82). PDX shares high concordance with the native tumor in terms of gene expression profiling, genetic mutation, and histopathology properties. However, the rate of success engraftment is only one out of 17 tumors (5.8%), and it is time-consuming with a growth latency of 4 months for the first generation. Want et al. reported their establishment of the CCA PDX mouse model using freshly resected tissues of metastatic lung nodules of CCA patients bearing FGFR2-CCD6 fusion protein (83). Using this model, they found that FGFR inhibitors could significantly inhibit tumor growth, and no synergistic effect was observed when ponatinib was used in combination with gemcitabine and cisplatin chemotherapy in this PDX model. Another study also reported that JQ1, an inhibitor of the bromodomain and extra-terminal domain (BET), could suppress tumor growth through Myc-inhibition-caused DNA damage and cell apoptosis in the PDX model (84). It is difficult to carry out high throughput drug screening in PDX models because of its high cost. Recently, Vaeteewoottacharn et al. reported their establishment of PDX-derived cell lines, which retains some degree of key characteristics of original tumors, can be used as a tool to carry out larger scale of drug screening (85). Despite the advantage of mimicking the biological microenvironment of human tumor better than *in vitro* 2D culture, wide application of PDX in the clinic was limited for its several drawbacks, such as low success rate of engraftment, time and resource consuming, not suitable for exploring the mechanisms of immunotherapy resistance, and the problem of mouse-specific tumor genetic evolution after serial transplant into immunodeficiency mouse (86, 87). It seems the combination strategies that PDO-based high throughput drug screening and further verification *in vivo* using PDO-derived xenograft models are more feasible in clinical application.

### Clinical Models for CCA

Like cell line models that identify drug resistance-associated genes through genetically comparing wild-type cell lines to artificially-established drug-resistant cell lines, clinical models compare genes expression in pre-treatment tissue biopsy with that in post-treatment tumor biopsy from patients exhibiting disease progression after drug administration. Using a clinical model, Krook et al. identified the role of secondary FGFR2 kinase domain mutation in acquired resistance to FGFR inhibitor infigratinib (88). Before infigratinib treatment, the patient underwent an ultrasound-guided tumor biopsy and next-generation sequencing (NGS). Despite the partial response at a time point of four months after infigratinib treatment, the patient exhibited disease progress eight months later, and a repeat biopsy and NGS of the progressive tumor was conducted. Comparing the differentially expressed genes in these two biopsies shed light on molecular mechanisms contributing to acquired infigratinib resistance. This kind of model can help guide individual medication. There are some disadvantages of this model, such as difficulties in obtaining pre-treatment and post-progression tumor biopsy, and the limitations of tumor biopsies in capturing tumor heterogeneity.

Although it is hard for these research models to frame the whole landscape of drug resistance in CCA patients for their not being able to completely recapitulate the complex microenvironment of patients, these preclinical research models are still widely applicated in the research of drug resistance. Using these models, a great contribution has been made to better understand the mechanism of drug resistance in CCA, especially regarding the role of adaptive change and intercellular communication of cancer cells in drug resistance. In the following part, we will discuss the mechanism of drug resistance in CCA.

## Mechanism of Drug Resistance in CCA

An R0 resection is the only curative strategy for CCA patients (89). However, patients often have tumor recurrence and metastasis, and the 5-year tumor-recurrence free survival rate for CCA patients after R0 resection is only 10–31% (90). Recently, several clinical studies suggested that postoperative adjuvant chemotherapy can improve the survival rate in CCA patients (91), and adjuvant capecitabine was recommended as the standard treatment for biliary tract cancer patients after surgery (92). Besides, great efforts have been made to investigate effective targeting therapies for CCA. However, the number of patients who benefit from adjuvant therapies is low, due to the intrinsic or obtained drug resistance after treatment. The complex mechanisms of chemoresistance (MOC) in CCA help cancer cells escape from the effect of anti-cancer agents, leading to the poor response of CCA to these therapies. Better understanding of the underlying mechanism for drug resistance may promote the establishment of novel combination therapies for CCA. The mechanisms contributing to drug resistance in CCA were previously divided into the following five groups: a decreased drug uptake and increased drug export; reduced intracellular activation of prodrugs or enhanced inactivation of active drugs; adaptive changes in the molecular targets; enhanced ability to repair drug-induced effects on target; activation of an anti-apoptotic signaling pathway or inactivation of a pro-apoptotic signaling pathway. Recently, the role of TME and phenotype transition of tumor cells in drug resistance of tumor was reported; however, their actual function in CCA drug resistance remains to be further explored. Here, we summarized the main mechanisms contributing to drug resistance in CCA.

### Decreased Drug Uptake and Increased Drug Export

Generally, anti-cancer drugs are transported intracellularly by plasma membrane transporters that belong to the solute carrier (SLC) superfamily of proteins (93), and the active drugs are exported out of cancer cells through several members of the families of ATP-binding cassette (ABC) proteins, such as ABCB, ABCC and ABCG. Both downregulated expression or impaired function of SLC protein and increased expression or enhanced function of ABC family in CCA reduce intracellular concentration of active drugs, thereby causing drug resistance (94).

The expression of the copper transporter (CTR1) (SLC31A1), a transporter of platinum derivatives, is significantly downregulated in CCA, which might result in reduced chemosensitivity to these drugs (95). It has been reported that CTR1 may act as a biomarker for treatment response to gemcitabine–platinum combination therapy in biliary tract cancer patients (96). The organic cation transporter 1 (OCT1) (SLC22A1), a functional transporter at the plasma membrane downregulated in CCA, contributes to sorafenib resistance in CCA (80, 97), leading to tumor progression and poor prognosis in human cholangiocarcinoma (98). Organic anion-transporting polypeptides A2 (OATP1A2) is a plasma membrane transporter mediating the cellular uptake of several anti-cancer drugs, such as methotrexate, taxanes and imatinib (99). CCAs express a low level of OATP1A2 (100), which plays a role in the reduced sensitivity to these drugs in CCA. Gemcitabine is one of the most efficient agents in the treatment of CCA, and the combined treatment of gemcitabine with cisplatin has been regarded as standard care for advanced CCA (10). However, CCA acquires resistance to gemcitabine through undergoing downregulation of equilibrative nucleoside transporters (ENTs) which is associated with gemcitabine uptake (101), and low expression of ENT1 is a biomarker for chemoresistance to gemcitabine in CCA patients (102, 103). Moreover, downregulation of ENT1 may also participate in 5-fluorouracil (5-F) resistance in CCA (104).

To reduce drug uptake, the intracellular concentration of active drugs can also be influenced by increased efflux of drugs mediated by ABC family proteins, such as ABCB, ABCC and ABCG (105). These proteins are expressed in normal cholangiocytes and play multifunction in the physiological activity of these cells. In the course of tumorigenesis, the expression levels of ABC family proteins are significantly upregulated in CCA. Moreover, ABC family proteins expression can be further upregulated after adaption to drugs treatment. For example, inducible Thymosin β10 overexpression after 5-FU treatment contributes to the resistance to 5-FU, with the underlying mechanism that Thymosin β10 activates the expression of ABC transporters (ABCB1, ABCG2) to export 5-FU (106). ABCB1 (P-glycoprotein or MDR1) is a member of the ABCB family located at the plasma membrane. ABCB1 participates in the transportation of anticancer drugs, such as doxorubicin, etoposide, paclitaxel, and vinblastine (107, 108), playing an essential role in the multidrug resistance phenotype of cancer cells (109). The higher expression of ABCB1 in CCA was observed (110). NF-kB and Notch1 might be upstream regulators of ABCB1 in CCA, for the observation that NF-kB and Notch1 inhibitor suppresses the expression of ABCB1and further enhances sensitivity to anticancer drugs (31, 110).

Multidrug resistance-associated proteins (MRPs) are transporters belonging to the ABCC family. Upregulation of MRP1 is found in gemcitabine-resistant CCA cell lines established by stepwise exposure to increasing concentration of gemcitabine, and the sensitivity of these cells to gemcitabine was recovered after silencing MRP1 (111). MRP3, another member of the ABCC family that is highly expressed in CCA cells, is closely associated with the IC50 values of etoposide, doxorubicin and pirarubicin, suggesting the potential role of MRP3 in resistance to these agents in CCA patients (110). A recent study has found that SOX17 is a negative regulator of MRP3, and MRP3 overexpression in CCA might be a result of low expression of SOX17 (112). SOX17 overexpression inhibited the expression of MRP3, potentiating the cytotoxicity of SN-38, 5-fluorouracil (5-FU) and mitoxantrone in CCA (112).

ABCG2, a member of the ABCG family, is another bump located at the plasma membrane and plays a significant role in chemotherapeutic resistance in various cancers through efflux of anticancer drugs, such as doxorubicin (113), methotrexate (114), imatinib (115), and irinotecan (116). Chen et al. reported that ABCG2 might be a downstream target of amplified in breast cancer 1 (AIB1) (117). AIB1 is overexpressed in CCA, and AIB1-induced ABCG2 overexpression plays a vital role in promoting drug efflux, contributing to enhanced chemoresistance in human cholangiocarcinoma (117).

### Reduced Proportion of Active Drugs in Cells

Some drugs are transported into cells in an inactivated form of prodrugs that need to be further activated by cellular enzymes, and the loss of these enzymes will cause drug resistance. For example, orotate phosphoribosyl transferase (OPRT) is associated with activation of 5-FU into FdUMP, and a reduced expression of OPRT has been found to be associated with poor response to 5-FU in CCA, suggesting that OPRT could be used as a predictor for the response to 5-FU in CCA (118). Besides, drug resistance may also be a result of the inactivation of drugs caused by detoxifying enzymes expressed in cancer cells, such asase I enzymes- NAD(P)H-quinone oxidoreductase 1 (NQO1), and phase II enzymes-placental (P) isoform of glutathione-S-transferase (GSTP1). In CCA, enhanced NQO1 activity is associated with chemoresistance to doxorubicin, 5-FU, and gemcitabine (119). β-eudesmol-mediated inhibition of NQO1 enhanced sensitivity to 5‐FU and doxorubicin in CCA (120). Cytochrome P450-related enzymes (CYP)s are a group of enzymes catalyzing the oxidation of compounds, CYPs participate in 80–90% of the phase I reactions associated with drug metabolism (121). CYP3A4 might be associated with erlotinib, sunitinib and sorafenib resistance in CCA (122). However, the other number of function of the CYP family in CCA drug resistance remains to be elusive. Conjugation with glutathione by GSTP1 plays a significant role in cellular neutralizing toxic compounds, including many anticancer drugs, such as platinum-derivatives (123). GSTP1 expression is upregulated in CCA, which may contribute to resistance to these drugs in CCA (124). The inhibition of expression or function of GSTP1 can sensitize CCA to doxorubicin, cisplatin, and several alkylating agents (124). Metallothioneins, enzymes able to neutralize irinotecan and platinum‐derived drugs, are overexpressed in CCA and have been observed to be predictors for the poor response of patients to chemotherapy based on platinum derivatives (125).

### Changes in Drug Molecular Targets

CCAs are characterized by high interpatient and intratumor heterogeneity, which makes it difficult to overcome the problem of Darwinian selection of most resistant subclones. Drug treatment pressure may elicit subclones that are naturally resistant to drugs after eliminating the sensitive cells. For example, drug-induced selection of tumor subclones with tyrosine kinase domain mutation is the main mechanism of resistance to BGJ398 in CCA patients carrying FGFR2 fusions (16). Cancer cells, originally sensitive to agents, can also become resistant to drugs after the selective pressures-induced changes in the molecular targets. For example, CCA patients, showing partial response during the first several rounds of treatment of FGFR inhibitor infigratinib, exhibited the secondary FGFR2 kinase domain mutation and become resistant to infigratinib after prolonged treatment (88). Increased expression of therapeutic targets may overcome the inhibitory effect induced by targeted agents. For example, CCA cells could upregulate the expression of EGFR after exposure to EGFR inhibitor erlotinib, becoming more resistant to apoptosis induced by erlotinib (126). Moreover, the downstream proteins of a therapeutic target can also undergo mutation to contribute to resistance to target therapy. For example, mutation of BRAF and KRAS (downstream targets of EGFR), which is frequent in CCA, contributes to resistance to anti-EGFR therapies (127). Recently, the CCA PDX model with KRAS mutation has been established, which might be a good model to further investigate the role of KRAS mutation in resistance to anti-EGFR therapy (82). In addition, an alternative signaling pathway can also be utilized by cancer cells to overcome the selective pressure of targeted agents. For example, after long-term exposure to erlotinib, CCA cells undergo upregulation of IGF2/IR/IGF1R signaling pathway, which contributes to resistance to erlotinib (17).

### Enhanced Ability to Repair Drug-Induced DNA Alterations

The cytotoxic effects of several drugs, such as cisplatin and 5‐FU, are achieved through their direct or indirect interaction with DNA structure to damage the genetic material of dividing cells. Cells, sensitive to these drugs, activate apoptosis signaling under the selective pressure of these drugs; whereas cells, able to repair drug-induced DNA alterations, can become resistant to these drugs. Commonly, the DNA repair can be attributed to specific DNA-repairing enzymes or several protein cascades involved in enzymatic repair systems, such as nucleotide- and base-excision repair (NER, BER), DNA mismatch repair (MMR), and recombination repair. The downstream target of p53, p53R2, is a ribonucleotide reductase associated with repairing damaged DNA, and the increased expression of p53R2 has been found to be involved in gemcitabine-resistance in CCA (128). Excision repair cross‐complementing 1 protein (ERCC1), a significant component of the NER system, can remove various bulky DNA adducts generated by cisplatin and alkylating agents. The clinical evidence regarding the role of ERCC1 in poor response to cisplatin has been revealed (129). ERCC1-negative patients benefit from cisplatin treatment; whereas limited benefits were observed in ERCC1-positive patients, suggesting the prognostic value of ERCC1 in CCA patients treated with cisplatin. The cisplatin-induced DNA damage can also be repaired by Tousled-like kinase 1 (TLK1) overexpressed in CCA, a serine/threonine proteins kinase regulating chromatin assembly and DNA repair pathway (130). Uracil-DNA glycosylase 1 (UNG1), initiator of the BER system, is significantly upregulated in 5-FU resistant CCA cell lines and plays an essential role in resistance to 5-FU *via* the repair of 5-FU induced DNA lesion (104). RAD51 protein plays a significant role in homologous recombination that can effectively repair DNA double-strand breaks caused by anticancer agents. In, breast cancer, RAD51 was found to be associated with poor response to anticancer agents, such as cyclophosphamide, epirubicin and docetaxel (131). Obama et al. has reported that RAD51 is upregulated in CCA, suggesting the potential resistance to these drugs in CCA with high expression of RAD51 (132).

### Re-Balancing Anti-Apoptotic and Pro-Apoptotic Factors

Cancer cells can escape from the drug-induced apoptosis *via* downregulation of pro-apoptotic mediators or upregulation of anti-apoptotic factors, exhibiting resistance to these drugs. In CCAs, high expression levels of Bcl2 and low expression levels of Bax have been associated with resistance to cisplatin and 5-FU (133).

Several pro-apoptotic genes, such as Bax, Bak, caspase 3, and caspase 9, have been reported to be associated with response to chemotherapy in CCA. Inactivating mutation or impaired expression of these genes contribute to poor response to chemotherapies. Tumor necrosis factor-related apoptosis-inducing ligand (TRAIL) belongs to the extrinsic pathway of apoptosis and is a promising therapeutic target for CCA (134). However, the increased expression of miR-25 in CCA can inhibit the expression of the TRAIL death receptor (DR4), causing the poor response of CCAs to TRAIL-induced apoptosis (135). The p53 protein is a significant regulator in the balance of anti-/pro-apoptosis, and the mutation of p53 is regarded as a predictor for a poor outcome in CCA (136). In addition, increased expression of anti-apoptotic factors, such as Bcl2 and survivin, also confers CCA enhanced resistance to anticancer chemotherapies (137). Nuclear factor erythroid 2-related factor 2 (Nrf2) is a transcription factor involved in regulating antioxidants and plays a cytoprotective role in drug-induced apoptosis (138). Several evidences have suggested the contribution of Nrf2 to chemoresistance of CCA partially for their role in regulating the expression of NQO1 and ABCC2, and maintaining the function of mitochondrial (139–141). Overactivation of several oncogenic signaling pathways favoring cell survival, such as PI3K/Akt, Raf/Mek/Erk, also contributes to chemoresistance in CCA and are summarized in the following part.

### Overactivation of Oncogenic Signaling Pathway

**Figure 2:**
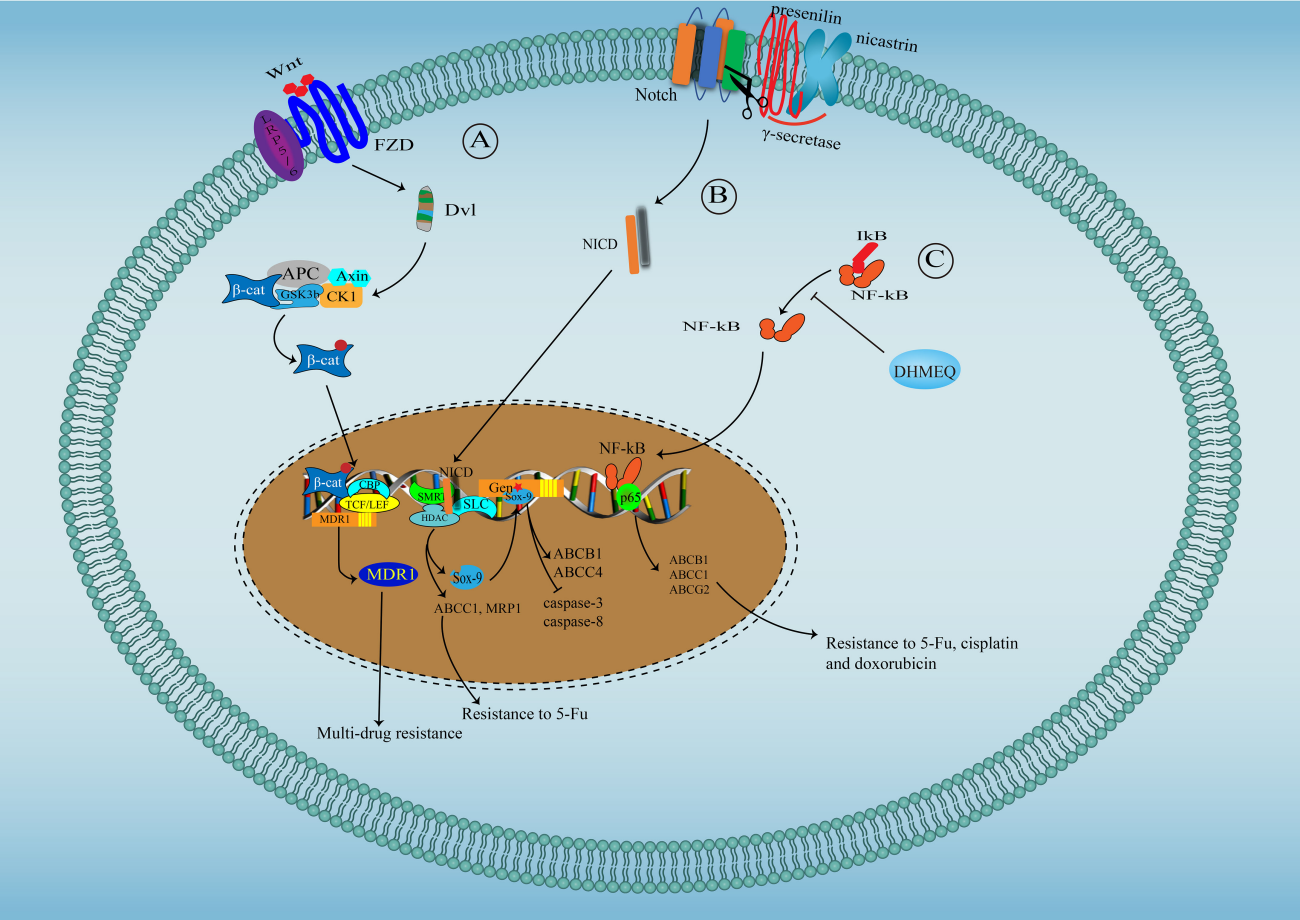
Signaling pathways involved in drug resistance in cholangiocarcinoma. **(A)** Wnt/β-catenin signal pathway in drug resistance in cholangiocarcinoma: Wnt binds to its receptor-Frizzled to activate Dsh protein, which phosphorylate and inactivate GSK3β, facilitating the translocation of free and unphosphorylated β-catenin from the cytoplasm to the nucleus, where β-catenin binds to TCF/LEF to promote MDR1 expression. **(B)** Notch signal pathway in drug resistance in cholangiocarcinoma: After Notch activation, γ-secretase (Presenilin and Nicastrin) cleaves Notch COOH-terminal fragment. NICD, released into the cytoplasm, further translocate to the nucleus, where NICD interact with SKIP and CSL, leading to SMRT/HDACs dissociation and converting CSL to a transcriptional activator to initiate the expression of ABCC1, MRP1 and Sox9, which can further promote the expression of ABCB1 and ABCC4. **(C)** NF-kB signaling pathway in drug resistance in cholangiocarcinoma: NF-kB translocate into the nuclear to initiate the expression of ABCB1, ABCC1 and ABCG2.

Several oncogenic signaling pathways, critical in tumor progression, also participate in the regulation of chemoresistance in CCA (**Figure 2**). Yes-associated protein (YAP)/Hippo pathway is a vital developmental pathway in maintaining stem cell properties and plays a significant role in resistance to apoptosis (142). YAP activity is significantly enhanced in CCA and has been associated with chemoresistance for its negative regulation of TRAIL, a key cancer cells death inducer (143). Both Akt phosphorylation and Erk1/2 phosphorylation are significantly increased in cisplatin-resistant CCA cell lines undergoing long-term exposure to cisplatin, and inhibiting Akt and Erk1/2 activation reverse chemoresistance to cisplatin, suggesting the contribution of Akt and Erk1/2 in resistance to cisplatin in CCA (133). Besides, overactivation of Akt has also been associated with resistance to fibroblast growth factor receptor (FGFR) inhibitor BGJ398 (144). Wnt/β-catenin signal is vital for stemness properties in cancer, and several studies have suggested that Wnt/β-catenin signal might be an ideal therapeutic target for reversing multidrug resistance in CCA for the observation that enhanced activity of Wnt/β-catenin signal contributes to multi-drug resistance phenotype of CCA through upregulating MDR1 (145). β-escin-induced inhibition of the β-catenin pathway can reverse the multidrug resistance of CCA (146). The Notch signaling pathway has been found to play a critical role in tumor development (147). Marin et al. have reported that Notch signaling, overactivated in CCA, can increase the expression of ABCC1 and MRP1, causing the poor response of CCA to 5-FU (31). Sex determining region Y-box (Sox)-9, a downstream target of Notch, is essential for stemness properties and resistance to gemcitabine in CCA. Mechanically, Sox9 promotes the expression of ABCB1 and ABCC4 to enhance the efflux of drugs, decreasing the expression of cleaved caspase-3 and caspase-8 induced by gemcitabine (148). NF-kB signaling, constitutively activated in CCA, has been shown to play a role in the resistance of CCA to 5-FU possibly through regulating the expression of ABCB1, ABCC1 and ABCG2 (149).

### Microenvironment-Mediated Chemoresistance in CCA

The tumor microenvironment (TME), composed of fibroblast, endothelial cells, immune cells, and extracellular matrix, represents an important component of tumor growth and progression. CCA is characterized by rich stromal, where cancer-associated fibroblasts (CAF) and extracellular matrix play significant roles in promoting CCA progression (150, 151). Recent pieces of evidence support the viewpoint that TME exerts effects in decreased sensitivity of CCA to chemotherapies, leading to the poor clinical outcome of CCA patients. In CCA, the increased expression of cytokine leukemia inhibitory factor (LIF), an inflammatory factor, contributes to resistance to chemotherapies (152). The hypoxia in TME also represents a significant cause of therapeutic resistance in solid tumors. Silakit et al. reported that hypoxia-induced overexpression of miR-210 contributes to chemoresistance to gemcitabine in CCA, where the proliferation inhibition and cell cycle arrest caused by miR-210 plays a significant role (153). Choodetwattana et al. have found that acidic extracellular pH performs a critical function in resistance to gemcitabine (154). The Octamer-binding transcription factor 4 (Oct4) transcription factor is upregulated in acidic extracellular pH-induced gemcitabine resistant CCA cell lines, suggesting a potential role of OCT4 in chemoresistance in CCA. More direct evidence regarding the role of OCT4 in acidic extracellular pH-induced chemoresistance in CCA needs to be provided by further studies. Cancer cells are characterized by rapid proliferation, and cancer tissues are often in shortage of oxygen and nutrients, including glucose (155). A recent study has revealed that CCA cells, adapted to the glucose depletion microenvironment, show enhanced stemness properties and acquire resistance to gemcitabine through reactive oxygen species ROS-mediated activation of Akt (156). Mesenchymal stem cells (MSCs) are multipotent progenitor cells and can be recruited to tumor sites to facilitate tumor progression (157). It has been reported that MSCs can interact with CCA cells and enhance Wnt/β-catenin signaling pathway activity in CCA, contributing to resistance to ginsenoside metabolite-compound K (158).

## Perspectives and Future Directions

Despite the progress in the development of experimental models and the great efforts devoted to developing new targeted therapies in CCA, the clinical outcomes of CCA patients remain to be poor, mainly for the challenge of translation of basic science to the clinical setting, and the intrinsic or acquired resistance to the therapeutic agents. Here, we have summarized the different experimental models available for CCA research. All these models have their advantages and drawbacks. Despite the apparent drawbacks, the 2D cell line model and cell line-based xenograft animal model are the most frequently used models for their being easy to operate and not costly. Novel technologies, such as CRISPR/CAS9 library and high-throughput drug screening, could be applied in these models to help identify critical genes implicated in drug resistance, favoring the development of combination treatment therapeutic strategies for CCA patients. Single-cell RNA-seq technology greatly enriched our knowledge of the heterogeneous property of tumors, and tumor heterogeneity was regarded as an important cause for treatment resistance (54). Theoretically, experimental models that can maintain the heterogeneous property of primary tumors can better recapitulate the drug response of CCA patients. Compared with cancer cell lines, PDO and PDX models can better recapitulate the pathohistological and genetic features of primary tumor tissue, thus better mimicking the response to the therapeutic treatment. This makes PDO and PDX models become a hot area of research. Several studies have already reported that PDOs can be used to predict responses in cancer patient in the clinic (159, 160). The model that uses the PDO model to conduct high-throughput drug screening and further confirm the response to agents in PDX seems to be promising in facilitating personalized medicine. High-throughput drug screening can also be used to evaluate the treatment response of combination therapy in PDO models, and this might clue us in on developing novel therapeutic strategies for CCA patients. However, there are some drawbacks to these two models. One obstacle for PDO and PDX models to better recapitulate primary cancer is not being able to simulate the tumor microenvironment in the primary tumor, especially the immune microenvironment. Recently, immunotherapy has dramatically changed the current standard of care in several cancers. In some cases, immunotherapy-based combination therapy could transform unresectable cancer into a curable disease (161, 162). Limited benefits were observed in CCA patients receiving monotherapy with an immune checkpoint inhibitor (163). However, the AstraZeneca company announced a result from the recent TOPZA-1 clinical trial that Imfinzi plus chemotherapy showed a statistically significant and clinically meaningful overall survival (OS) benefit versus chemotherapy alone in CCA patients, and this may change the current standard of first-line treatment for CCA patients. Preclinical experiment models that could help uncover the mechanisms of immunotherapy resistance or imitate response to immunotherapies in CCA patients will further improve the clinical benefits of immunotherapy. A recent study reported an organoid model of the tumor immune microenvironment containing primary tumor epithelium and endogenous immune stroma, and this model may facilitate personalized immunotherapy testing (164). Schnalzger et al. reported a 3D model for CAR-mediated cytotoxicity using PDOs, a sensitive platform for evaluating CAR efficiency in a personalized manner (165). It is possible that PDO could also be used to predict the response to immunotherapies in CCA patients. Although the PDX model can recapitulate the heterogeneity and the tumor microenvironment in primary tumor, it cannot mimic the immune microenvironment. So, it is difficult to do researches regarding immunotherapies in CCA using this model. Humanized mice, that could bear cancer cells and immune cells from human at the same time might be used with the PDX model to offset the drawbacks of PDX models, creating an excellent preclinical model that could rapidly and safely allow us to explore the underlying mechanisms of sensitivity and prediction of immunotherapies of the response to immunotherapy of CCA patients (166). We believe that preclinical study models for cancer research will become more perfect with the development of technology.

The resistance to anticancer treatment is another reason for the dilemma in CCA treatment. A better understanding of the molecular mechanisms underlying the poor response of CCA to anticancer treatment will facilitate the identification of therapeutic targets for improving the efficiency of anticancer treatments. Several mechanisms have been associated with chemoresistance in CCA: reducing import of drugs or increasing efflux of drugs; inactivation of drugs; changes in drug molecular targets; enhanced ability to repair drug-induced DNA alterations; re-balancing anti-apoptotic and pro-apoptotic factors; overactivation of oncogenic signaling pathway; pro-survival microenvironment induced by cancer cells. Combination therapies that reverse the mechanisms underlying multidrug resistance in CCA seem to be a promising therapeutic strategy. For example, to enhance intracellular drug concentration, several strategies have been proposed: combined usage of anticancer agents with compounds that alter membrane fluidity of cancer cells to facilitate anticancer drug influx (167); encapsulation of drugs into liposomes or nanoparticles that facilitate drugs uptake (168, 169); gene therapies enhancing the expression of drugs uptake transporters (170); chemo-sensitizing agents inhibiting the functions of ABC pump (171). MDR may also be overcome by targeted therapies that inhibit oncogenic signaling pathways overactivated in resistant CCA cells, such as Wnt/β-catenin signaling (145), NF-kB signaling (149) and PI3K/Akt/mTOR signaling pathway (172). Basic scientific research has identified various therapeutic targets for CCA, such as genetic aberrations, TME, melatonin and circadian rhythms, and non-coding RNAs that play a significant role in CCA progression (reviewed elsewhere) (7). Based on these targets, the development of more efficient anticancer agents and therapeutic strategies will help overcome the dilemma in CCA treatment.

## Author Contributions

All authors listed have made a substantial, direct, and intellectual contribution to the work and approved it for publication.

## Conflict of Interest

The authors declare that the research was conducted in the absence of any commercial or financial relationships that could be construed as a potential conflict of interest.

## Publisher’s Note

All claims expressed in this article are solely those of the authors and do not necessarily represent those of their affiliated organizations, or those of the publisher, the editors and the reviewers. Any product that may be evaluated in this article, or claim that may be made by its manufacturer, is not guaranteed or endorsed by the publisher.
